# Biochar Addition Alters C: N: P Stoichiometry in Moss Crust-Soil Continuum in Gurbantünggüt Desert

**DOI:** 10.3390/plants11060814

**Published:** 2022-03-18

**Authors:** Yaobao Chang, Weiguo Liu, Yuqing Mao, Tao Yang, Yinguang Chen

**Affiliations:** 1 College of Ecology and Environment, Xinjiang University, Urumqi 830017, China; byy042627@xju.stu.edu.cn (Y.C.); myq@xju.stu.edu.cn (Y.M.); tyang165@xju.stu.edu.cn (T.Y.); 2 Key Laboratory of Oasis Ecology of Education Ministry, Urumqi 830017, China; 3 Xinjiang Jinghe Observation and Research Station of Temperate Desert Ecosystem, Ministry of Education, Urumqi 830017, China; 4 School of Environment Science and Engineering, Tongji University, Shanghai 200092, China

**Keywords:** local plant biochar, moss crusts, nutrient cycling, ecological stoichiometry, desert ecosystems

## Abstract

The biogeochemical cycling of soil elements in ecosystems has changed under global changes, including nutrients essential for plant growth. The application of biochar can improve the utilization of soil nutrients by plants and change the stoichiometry of carbon (C), nitrogen (N), and phosphorus (P) in plants and soil. However, the response of ecological stoichiometry in a moss crust-soil continuum to local plant biochar addition in a desert ecosystem has not been comprehensively explored. Here, we conducted a four-level *Seriphidium terrae-albae* biochar addition experiment (CK, 0 t ha^−1^; T1, 3.185 t ha^−1^; T2, 6.37 t ha^−1^; T3, 12.74 t ha^−1^) to elucidate the influence of biochar input on C: N: P stoichiometry in moss crusts (surface) and their underlying soil (subsurface). The results showed that biochar addition significantly affected the C, N, and P both of moss crusts and their underlying soil (*p* < 0.001). Biochar addition increased soil C, N, and P concentrations, and the soil N content showed a monthly trend in T3. The C, N, and P concentrations of moss crusts increased with the addition levels of biochar, and the moss crust P concentrations showed an overall increasing trend by the month. Moreover, the soil and moss crust C: P and N: P ratios both increased. There was a significant correlation between moss crust C, N, and P and soil C and N. Additionally, nitrate nitrogen (NO^3−^N), N: P, C: P, EC, pH, soil moisture content (SMC), and N have significant effects on the C, N, and P of moss crusts in turn. This study revealed the contribution of biochar to the nutrient cycle of desert system plants and their underlying soil from the perspective of stoichiometric characteristics, which is a supplement to the theory of plant soil nutrition in desert ecosystems.

## 1. Introduction

Carbon (C), nitrogen (N), and phosphorus (P), the three most basic elements in soil, play crucial roles in organismic activities [1]. C is a key building block of structural substances, whereas N and P are the main limiting nutrients for the productivity of terrestrial ecosystems [2,3]. In nutrient-deficient areas, plants need to regulate the stoichiometry of C, N, and P in the plant-soil system to maximize the utilization of soil nutrients in confronting the adverse environment [4]. Exploring the relationship in C, N, and P stoichiometry between plants and soil is important for the understanding of biogeochemical cycles in terrestrial ecosystems [5,6]. Many studies have reported the C, N, and P stoichiometry of plants and soil, and analyzed their relations and influence factors [7,8], most studies, however, have mainly focused on forest and steppe ecosystems, while neglecting desert ecosystems [5,9].

Biochar is a kind of charcoal produced by burning biomass (organic material) in a low-oxygen environment. As its stable chemical properties contain many N, P, K and other nutrients necessary for plant growth and development [10], biochar is commonly used as a soil nutrient harmonizer [11]. Fertilizing biochar into soil also improves the utilization of soil nutrients by plants by improving soil structure, water, and fertilizer conservation capacity and microbial activities [12,13]. As a result, C, N, and P stoichiometry between soil and plants would be changed. For example, biochar addition decreases the C: N ratio in forest ecosystems by reducing the leaching of organic carbon and nitrogen [14]. Wood biochar increases nitrogen retention in field settings mainly through abiotic processes [15]. In addition, previous studies showed that the physical and chemical properties of biochar were related to its raw materials, and different types of biochar had different effects on plants and soils [16]. However, most of these studies have taken place in terrestrial wetter ecosystems, our study regarding the influence of biochar addition on C, N, and P stoichiometry in desert ecosystems is still not clear.

Biological soil crusts (BSCs) are composed of cyanobacteria, algae, fungi, lichens, bacteria, and mosses. As it can maintain surface stability, improve soil structure, affect soil nutrient cycling, and create favorable conditions for soil and phytoremediation of desert ecosystems, BSCs are considered to be an important basis for vegetation succession in arid and semi-arid desert regions [17,18,19]. The desert ecosystem is more barren than other ecosystems, plant growth is limited by nitrogen and phosphorus. The moss crusts are the most common form of all BSC types, accounting for about 50% of the total distribution area [20]. Due to its high ability to bind N, sequestrate C, and excite P, it is considered to be the largest contributor to improving desert surface nutrient cycling [21,22]. The input of exogenous nitrogen and phosphorus is beneficial to the growth of moss crusts, which can stimulate the photosynthetic process of moss crusts and enhance C exudate, thereby improving C: N: P stoichiometry [23]. However, most of the current studies have only focused on the positive maintenance of moss crusts on desert ecosystem functions, while few studies have investigated the effects of biochar addition on C: N: P stoichiometry in moss crust and their underlying soils (moss crust-soil continuum).

To verify the effects of biochar addition on C: N: P stoichiometry in the moss crust-soil continuum in desert ecosystems, we established a multi-level biochar addition indoor experiment using moss crusts and *Seriphidium terrae-albae* from the Gurbantünggüt Desert. The purposes of this study were: (1) to study the effects of biochar on C: N: P stoichiometry in the moss crust-soil continuum; (2) to identify the main regulatory factors affecting C: N: P stoichiometry in the moss crust-soil continuum. We applied local plant biochar to a desert ecosystem to change the C, N, and P of the moss crust system, and thereby stimulate the growth of moss crusts and other plants, which is of great significance for the improvement of structural stability and function of the desert ecosystem. Our results can provide a theoretical basis and data support for the application of biochar in global desert ecosystem restoration.

## 2. Materials and Methods

### 2.1. Study Site

The study site is located at the southern margin of the Gurbantünggüt Desert in the Xinjiang Uygur Autonomous Region, Northwest China (44°15′–46°50′ N, 84°50′–91°20′ E). This region has a typical temperate continental arid climate with a mean annual precipitation ranging from 70 to 150 mm, while the annual evaporation is greater than 2000 mm. The mean annual temperature ranged from 6 to 10 °C. The climate type of the region is BWk based on the Köppen climate classification [24]. The native vegetation is mainly composed of sand-borne and drought-tolerant plants. The dominant plant species include *Haloxylon ammodendron*, *Haloxylon persicum*, and some dwarf shrubs, specifically *Ephedra distachys*, *Calligonum mongolicum, Reaumuria soongorica, Artemisia arenaria,* and *Seriphidium terrae-albae*. In our study areas, moss crusts were 8–16 mm thick, biomass was 3.22–8.65 g dm^2^, and coverage was 61.2–86.8% [25].

### 2.2. Preparation of Biochar

According to our measurements and the references, *Seriphidium terrae-albae* has a relatively high dry biomass with the above-ground dry biomass being 54.6–82.2% of the total dry biomass in the plot. It is the most constructive species and dominant species in the habitat, widely distributed in semi-fixed or fixed sandy areas and forms a companion community with moss crusts [26,27]. Therefore, in this paper, the dominant species *Seriphidium terrae-albae* in the study area was selected as the biochar raw material. The carbon, nitrogen, and phosphorus contents of the biochar used in the experiment were, respectively, 71.6–82.3%, 0.92–1.17%, and 5.8–6.9 g/kg, and the pH was between 9.5–9.8. More specifically, the dried *Seriphidium terrae-albae* were pyrolyzed at 400 °C for 2 h under oxygen-limited conditions using vacuum tubular resistance furnace. After that, all samples were mixed, ground, and sieved with a soil sieve to particles <2 mm in diameter.

### 2.3. Experimental Design

To avoid disturbance from external factors such as animal and human activity damage, we conducted an indoor control experiment to test the influence of biochar addition on C: N: P stoichiometry. The amount of biochar used varies depending on its application, considering that the general biochar addition dosage range is 0–20 t ha^−1^, when it is used as a soil amendment in farmland ecosystem [28,29,30]. Therefore, our experiment included four biochar addition treatments: three biochar addition treatments (T1, 3.185 t ha^−1^; T2, 6.37 t ha^−1^; T3, 12.74 t ha^−1^) and a control (CK; 0 t ha^−1^). Moss crusts and soil (sandy soil) used for experiments were obtained from an experimental field in the southern margin of the Gurbantünggüt Desert, where long-term field experiments were conducted. To be specific, 12 plots (1 m × 1 m) were randomly selected in April 2018 in the sites where the moss crusts were well-developed and undisturbed. Moss crusts were collected in these plots using a soil column sampler (PVC pipe, d = 0.2 m) and a total of 12 soil columns. Then, all soil columns were buried separately in circular truncated cone stainless steel buckets containing situ soil (the lower opening of PVC pipe fits exactly into the bottom of the stainless teel buckets) and brought back to the laboratory for biochar addition experiments. Considering that desert ecosystems are almost unaffected by human and mechanical tilling, the biochar was uniformly spread on the surface (d = 0.2 m) of the moss crust at four levels (CK; T1; T2 and T3) at the end of May 2018, and each level was replicated three times [31]. All experimental treatments were placed in a plant culture room, and during the experiment the environmental conditions in plant culture room were set as follows: a day/night regime of 14 h light (7:30 a.m.–9:30 p.m.)/10 h dark, temperature 23 ± 1 °C, the daylight was set at 10,000 lx, and deionized water was sprayed on each experimental treatment with a spray bottle (twice a month on the 10th and 25th of each month, 10 mL each time, a total of 20 mL per month) according to the rainfall (the average annual precipitation is 70–150 mm) in the field.

### 2.4. Field Sampling and Measurement

**Figure 1:**
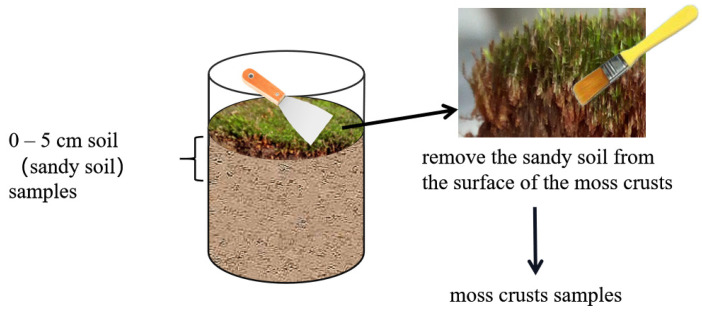
Schematic diagram of moss crust and soil sampling.

The moss crusts and soil samples were collected at the end of each month during the experiment period from June to October 2018 (Figure 1). Firstly, the moss crusts were carefully separated from the underlying soil using a small shovel to ensure the integrity of the moss crusts, and the sand soil from the surface of the moss crusts was gently removed with a brush. Then, moss crust samples were placed into a numbered envelope. After that, the subsoil (0–5 cm sandy soil) was collected where the moss crusts had been collected, and quickly brought to the laboratory for physical and chemical analysis [32].

The moss crust samples were dried for 48 h in an oven at 70 °C and screened by 2 mm mesh and then ground for determination of C, N, and P concentration. Soil samples were divided into two parts: one was used for soil moisture (SMC) determination using the oven drying method, and the other was air-dried at room temperature, hand-picked to remove plant and detritus, and ground to pass through a 100-mesh sieve for soil chemical determination.

### 2.5. Physicochemical Analysis

Various physico-chemical parameters were determined by adopting standard methods and all reagents used were from regular manufacturers. Total N concentration (TN) in the moss crusts and soil samples were determined following the Kjeldahl digestion method using a Nitrogen Analyzer System (KJELTEC 2300 AUTO SYSTEM II, Foss Tecator AB, Höganäs, Sweden) [33]. The total P concentration (TP) of the soil and moss crust samples was determined by the H_2_SO_4_–HClO_4_ digestion method and molybdenum blue method according to the Chinese standard (NY/T 88-1988) [34]. Soil organic C analysis used the potassium dichromate-sulfuric acid oxidation method according to the Chinese standard (HJ 695-2014) [35], while plant C concentration was measured by the dry combustion method using a multi N/C 2100 analyzer (Analytik, Jena, Germany). Soil pH was measured in a 1:2.5 mixture of soil and deionized water using a digital pH meter (PHS-3C, China) calibrated with a pH buffer of 4.01 and 6.86 pH, according to the Chinese standard (HJ 962-2018). The content of soil ammonium nitrogen (NH_4_^+^-N) and nitrate nitrogen (NO_3_^−^-N) was determined by the indophenol blue colorimetric method [36]. The determination of each sample was repeated three times.

### 2.6. Statistical Analysis

A one-way analysis of variance (ANOVA) was performed to test the effect of biochar addition on the nutrient traits (e.g., C, N, P) of the moss crusts and their underlying soils. Duncan’s honest significant difference test was used to assess the significant differences in the selected parameters among all of the treatments. The linear regression analysis was used to test the relationship between C, N, and P concentrations in the moss crusts and their underlying soils. Redundancy analysis (RDA) was used to explore the relationship between soil physicochemical characteristics (including SMC, pH, C, N, P, C: N, C: P, and N: P ratio, soil ammonium nitrogen and nitrate nitrogen) and moss crusts stoichiometric characteristics (C, N, P, C: N, C: P, and N: P ratios). Structure equation modeling (SEM) was used to explain how biochar addition influences nutrients of moss crusts and its underlying soil through hypothetical pathways. Statistical analyses were performed using R 4.0.2 software.

## 3. Results

### 3.1. Effect of Biochar Addition on Soil C, N, P Contents under Moss Crusts

In this study, the average soil OC, TN, and TP concentrations were 6.67 g kg^−1^ (range 3.93–9.54), 0.83 g kg^−1^ (range 0.5–1.2), and 0.36 g kg^−1^ (range 0.32–0.38), respectively. The soil C: N, C: P, and N: P ratio average levels varied from 7.86 to 8.33, 12.28–25.78, and 1.56 –3.24, respectively, the mean values of C: P and N: P both increased (Table 1).

**Figure 2:**
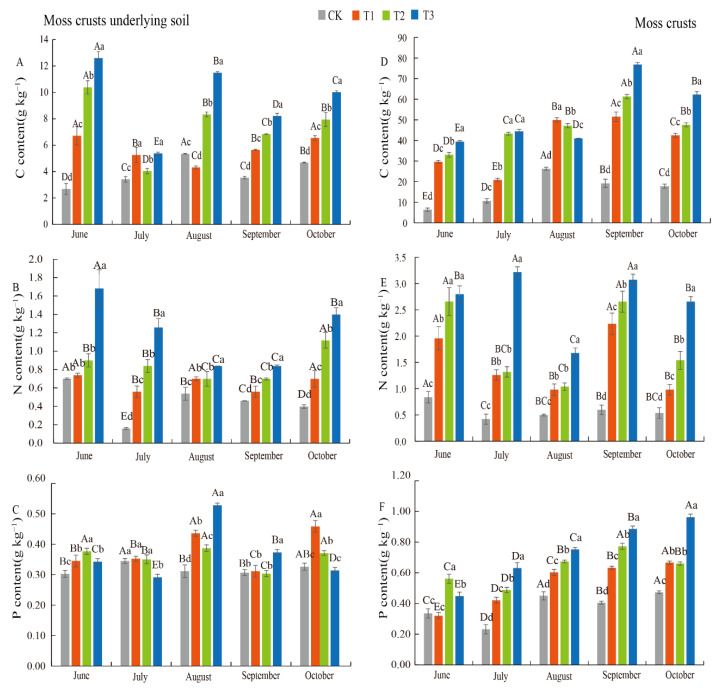
Changes of C, N, and P contents in moss crusts underlying soil (**A**–**C**) and moss crusts (**D**–**F**) with different levels of biochar addition. The C, N, and P contents are total forms, CK:0 t ha^−1^; T1: 3.185 t ha^−1^; T2: 6.37 t ha^−1^; T3: 12.74 t ha^−1^). Different capital letters indicate a significant difference among five sampling months with the same treatment; different lowercase letters indicate a significant difference among four biochar treatments in the same sampling period. Vertical bars show the standard error (SE) (*n* = 3).

The alterations of the C, N, and P concentrations in moss crust underlying soil from different treatments are shown in Figure 2. Our results showed that the addition of biochar increased the soil C, N, and P concentrations and reached the maximum at T3 treatment; moreover, the maximum value of C and N content was found in the T3 treatment in all experimental periods (from June to October). Meanwhile, the C content in June, September, and October and N content in all experimental periods significantly increased with the increase of the biochar addition levels (Figure 2A,B). In contrast, the P content varied in June, July, and October, during which P contents first increased and then decreased with the increase of biochar addition (Figure 2C). Moreover, the soil N content showed a clear “V” type characteristic by the month in T3 treatment.

### 3.2. Effect of Biochar Addition on Nutrient Traits of Moss Crusts

In this study, for treatment biochar addition, the C, N, and P concentrations of the moss crusts varied from 16.07 to 52.79 g kg^−1^, 0.58–2.7 g kg^−1^, and 0.35–0.74 g kg^−1^, respectively. Additionally, the moss crust C: N, C: P, and N: P ratio average levels varied from 19.55 to 27.71, 45.9–73.78, and 1.65–3.65, respectively, the mean values of C: N decreased (Table 2).

Biochar addition elevated the C, N, and P concentrations of the moss crusts (Figure 2). The C, N, and P increased with the increase of biochar addition, and the highest values were found in T3 treatment and the lowest was found in the CK. More specifically, the moss crust C content was significantly higher than the control in all treatments (Figure 2D). The N content continuously increased with the increase of biochar addition levels in all experimental period. The highest N content was observed in the T3 treatment in all months (Figure 2E). The P content showed a clear increasing trend with the increased addition of biochar and an overall increasing trend with the month (Figure 2F).

### 3.3. Nutrients Relationship between Moss Crusts and Its Underlying Soils with Biochar Addition

**Figure 3:**
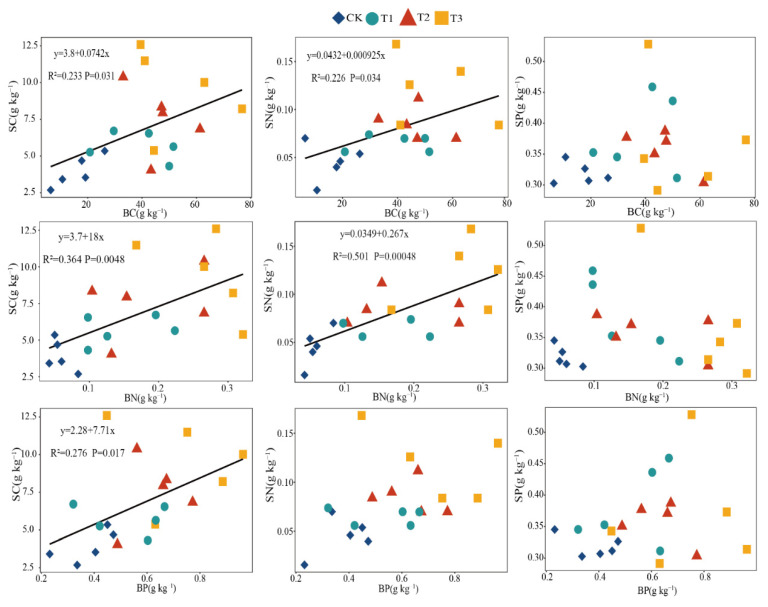
Linear regression indicating relationships between OC (C), TN (N), and TP (P) in the soil and C, N, and P in the moss crusts under different biochar treatments. The solid blank line indicates a significant correlation (*p* < 0.05). Notes: BC = moss crust C, BN = moss crust N, BP = moss crust P, SC = soil C, SN = soil N, SP = soil P.

Figure 3 showed the correlation analysis between the moss crust C, N, and P characteristics and their underlying soils nutrients, the C, N, and P of the moss crusts and the soil C, N, and P had a correlation under the biochar addition treatments. There were significant linear correlations between moss crust C and soil C, moss crust C and soil N, moss crust N and soil C, moss crust N and soil N, and moss crust P and soil C under the biochar addition treatments (*p* < 0.05). There was a nonlinear correlation between moss crust C, N, P and soil P, and moss crust P and soil N (*p* > 0.05).

**Figure 4:**
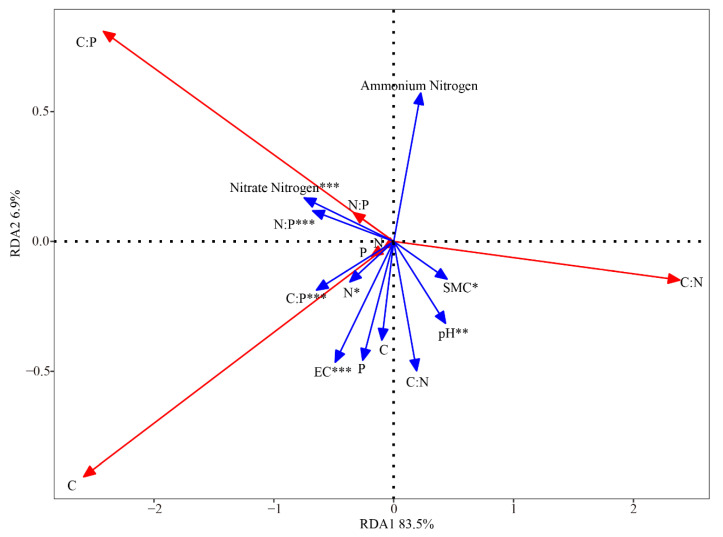
Redundancy analysis (RDA) of the moss crusts stoichiometric characteristics and its underlying soil physicochemical characteristics. In the biplot, the red lines in the figure represent the soil physicochemical characteristics, and the blue lines represent the moss crust C: N: P stoichiometry, respectively. Notes: * *p* < 0.05, ** *p* < 0.01, and *** *p* < 0.001, SMC = soil moisture content, EC = electrical conductivity.

We took moss crust organic carbon, total nitrogen, total phosphorus, C: N, C: P, and N: P as response variables, and soil C, N, P, C: P, C: N, N: P, Ammonium Nitrogen, Nitrate Nitrogen, pH, EC, and SMC as environment variables. Table 3 showed that compared with the control, biochar addition increased soil ammonium nitrogen, nitrate nitrogen, pH, EC, and SMC. Redundancy analysis (RDA) showed that the soil properties (C, N, P, C: P, C: N, N: P, Ammonium Nitrogen, Nitrate Nitrogen, pH, EC, and SMC) explained 90.4% of the total variation in the moss crust C, N, P content and C: N: P stoichiometry, with axes 1 and 2 explaining 83.5% and 6.9% of the total variation, respectively. The order of their effect on moss crusts were as follows: Nitrate Nitrogen, N: P, C: P, EC, pH, SMC, N, P, Ammonium Nitrogen, C: N, and C. Among them, nitrate nitrogen (NO_3_^−^-N), N: P, C: P, EC, pH, soil moisture content (SMC), and N have significant effects on the C, N, and P of moss crusts in turn (Figure 4, Table 4).

**Figure 5:**
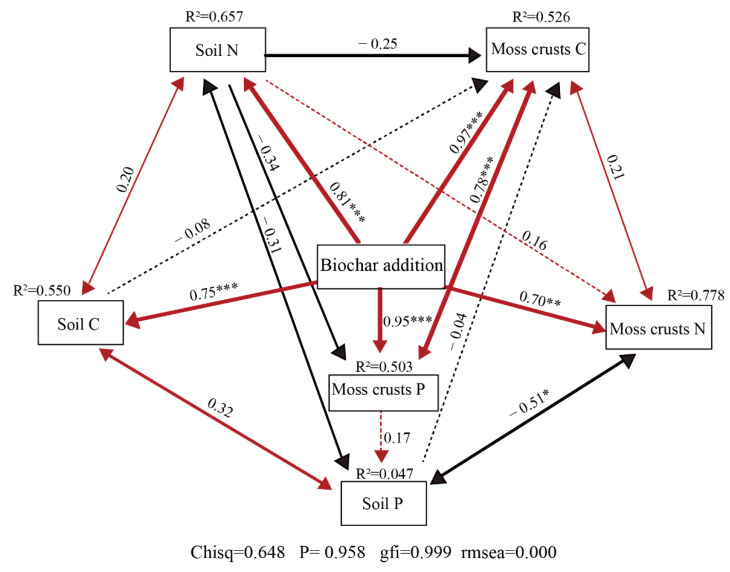
Structural equation model (SEMs) illustrating the effects of addition biochar on C, N, P of moss crusts and its underlying soil. Adjacent numbers that are labeled in the same direction as the arrow represents path coefficients, and the width of the arrow is in proportion to the degree of path coefficients. Blank and red arrows indicate negative and positive relationships, two ways arrows indicate mutual influence, continuous and dashed arrows represent the important and not important influence relationships, respectively. Significance levels are denoted with * *p* < 0.05, ** *p* < 0.01, and *** *p* < 0.001, The low chi-square (CMINDF), non-significant probability level (*p* > 0.05), high goodness-of-fit index (GFI > 0.90), and low root-mean-square errors of approximation (RMSEA) listed below the SEMs indicate that our data matches the hypothetical models. SMC = soil moisture content, R^2^ values indicate the proportion of variance explained by each variable.

Structure equation model (SEMs) analysis (Chisq = 0.648, P = 0.958, GFI = 0.999, RMSEA = 0.000) showed that biochar directly significantly affected C, N, and P both of moss crusts and their underlying soil (*p* < 0.001). The addition of biochar resulted in an interactive but non-significant (*p* > 0.05) effect between soil C, N, and P. Meanwhile, soil nutrient has almost no direct effect on moss crust C, N, and P, but what is interesting is that soil P was significantly negatively correlated with moss crust N. In addition, moss crust P was significantly correlated with moss crust C (Figure 5).

## 4. Discussion

### 4.1. Effect of Biochar Addition on Soil Nutrient Underlying Moss Crusts

Soil OC, TN, and TP contents are important indicators of soil nutrient status, which can determine the cycle and balance characteristics of C, N, and P in the soil, and evaluate C, N, and P mineralization, immobilization, and retention effects [37,38]. Previous studies have shown that the application of biochar can enhance soil water availability, water holding capacity, soil soluble organic carbon content, available phosphorus, total nitrogen, total organic carbon, soil microbial biomass carbon, nitrogen, and phosphorus (SMBC, SMBN, SMBP), soil microbial and nutrient retention and availability, which result in less fertilizer needs and reduce nutrient leaching [39,40,41].

In this study, we found that the high concentration of biochar addition could efficiently replenish soil nutrition, reduce the rate of soil nutrient release, and reduce nutrient loss [42]. More specifically, the content of C and N was related to the level of biochar addition; the more biochar added, the higher the C and N content, and the soil N showed a “V” type characteristic by the month; this is consistent with the results of the nitrogen addition experiment on C: N: P stoichiometry in moss crust-soil continuum [25]. It indicated that the nutrients from biochar contributed to the increases in C and N in the soil and biochar, which can effectively inhibit the leaching and migration of vegetation soil nitrogen [41]. Although one study found that there was no significant impact on the total soil nutrient by biochar addition, while organic carbon was significantly increased (*p* = 0.009) by 23% [43]. Compared to C and N, the change of soil P content was different, the soil P content increased first and then decreased but changed insignificantly with the addition of biochar. The positive effects of biochar on the soil ecosystem, including both plants and microbes, being proposed to derive either directly from nutrients within biochar itself, or indirectly from its ability to sorb and retain nutrients [44]. On the one hand, the effect of biochar addition on plant growth varies with type and dosage of biochar [16]. On the other hand, the community structure is directly associated with soil chemical properties, such as C or N levels [45], the addition of biochar increased the plant absorption of phosphorus by influencing the rhizosphere microorganism and increased the supply of available phosphorus in soil [4,46]. Other studies also found the content of soil C, N, and P in the whole growing season were 2.65–44.4, 0.02–0.32, and 0.3–0.80 g/kg after biochar addition [47]. In this study, during the study period, the soil C, N, and P contents were greater compared to CK, which indicated that adding a certain amount of biochar can improve the stability and nutrients of desert soil aggregates, enhancing life activities, which is conducive to the maintenance of soil fertility and sustainable and healthy development [48].

### 4.2. Response of Moss Crust C, N, and P Content and C: N: P Stoichiometry to Biochar Addition

BSCs can enhance the nutrient circulation of surrounding vascular plants through biological nitrogen fixation [19,49]. Based on biochar addition experiments results, our study demonstrated that biochar addition contributed to the increases in C, N, and P in the moss crusts. More specifically, the contents of C, N, and P in moss crusts increased with the increase in the amount of addition, which was similar to previous research results [13]. It indicated that the appropriate concentration of biochar addition could enhance the accumulation of N and the fixation of C, and possibly accelerate the progress of plant life activities, resulting in the accumulation of more nutrients [50]. The reasons for this may be the addition of biochar directly affects the microbial activity in the rhizosphere of the moss crusts to promote the conversion of nitrogen and phosphorus, thereby increasing the nitrogen and phosphorus content of the moss crust, and finally the C: N: P stoichiometric ratio of the moss crust is changed, and the C content also increases [51,52]. Furthermore, several studies have shown that biochar not only increased the K concentration in soil but also increased the plant nutrient use efficiency of K, which is important for plant growth. The above research results all showed that adding biochar enhances the ability of plants to absorb nutrients [53].

Carbon is the most important element that makes up the plant body, nitrogen and phosphorus is required for plant growth. It is widely reported that the N and P concentrations and N: P ratio can provide important information about nutrient limitation, an unbalanced input of N and P will seriously affect the ecological stoichiometry, ultimately affecting the function of the ecosystem [54]. The stoichiometry ratios of plant tissues vary with their growth and succession stages, and furthermore, the stoichiometry ratios, to some extent, can also reflect the rate of element absorption and utilization by plants [55]. For example, there is a strong correlation between C and N. The C: N indicates the ability of plants to absorb N. Studies have shown that the plant growth rate will be lower when the C: N value in the plant is higher. Our study also showed that the C: N of moss crusts decreased with the addition of biochar, this indicated that the higher concentration of addition can effectively promote the growth of moss crusts, biochar addition can effectively alleviate nitrogen limitation in desert ecosystems. In addition, biochar addition alleviates the negative effects on soybean productivity and water use efficiency under drought and salinity stress, thereby affecting crop growth [56]. All this indicated that the addition of biochar can improve the ability of moss crusts to resist adversity, promote the growth of surrounding vascular plants, the distribution of biological soil crusts (BSCs) has been confirmed in major desert regions of the world, the coverage of BSCs even reaches more than 70% in some deserts [27]. BSCs have very important ecological function for desert ecosystems, which can change the physical and chemical properties of the soil, improve the quality of the soil under drought conditions and enhance its stability [49], and the application of biochar is beneficial to the growth of BSCs, that is why biochar addition can improve the desert ecosystem.

### 4.3. Effect of Soil Factor on Moss Crusts Stoichiometric Characteristics with Biochar Addition

The ecological stoichiometry of plant was reported to not only depend on the biological characteristics of plants, but also have a strong correlation with soil factors [7,57]. Thus, studying the soil driving factors can further understand the interaction mechanism between plants and soil nutrients and reveal the interconnection and internal effects of biogeochemical cycles. In this study, moss crusts and their underlying soils were considered as an interactive system. It was found that there were significant linear correlations between moss crust C and soil C, moss crust C and soil N, moss crust N and soil C, moss crust N and soil N, and moss crust P and soil C under the biochar addition treatments (*p* < 0.05). In addition, the effect of biochar addition on moss crust C, N, and P contents was observed to be more significant than soil C, N, and P contents (Figure 4). One possible reason was that the biochar stimulated plant growth and its utilization of nutrients. Moss crusts could absorb water and nutrients and fixed C by photosynthesis to synthesize organic matter [58]. Adding biochar also facilitated moss crusts to release nutrients to the soil, which led to further changes in the soil and plants C: N: P stoichiometric ratios. The higher the content of C, N, P and available nutrients in the soil for plants, the stronger their growth and anti-interference ability, and the more complex the community structure [59].

Furthermore, RDA analysis showed that soil nitrate nitrogen was the key factor influencing the C, N, and P of moss crusts, followed by the N: P and C: P ratios, indicating that the supplement and transformation of N and P were crucial for the growth of plants in a barren ecosystem. Meanwhile, soil EC, pH, SMC and N also had significant effects on the stoichiometric characteristics of moss crusts. The possible reason was that biochar addition firstly changed soil environmental factors, such as soil pH and SMC of moss crust inter-roots, and then affected microbial activity, enhanced carbon exudation, and promoted the transport and transformation of nitrogen and phosphorus [22,60]. Previous studies have verified that biochar can promote the transport and transformation of N and P, and affect the diversity, activity, and abundance of nitrifier and denitrifier and Azotobacter in soil nitrogen cycling processes, which are directly related to a series of microbial activities in the nitrogen cycle [61,62]. In addition, biochar also participated in the phosphorus cycle in the soil ecosystem, increased the supply of available phosphorus in the soil, and had an important impact on the transformation process of soil phosphorus [63]. All of these indicated the importance of biochar addition to the desert ecosystem.

## 5. Conclusions

In summary, this paper investigated the changes and stoichiometric characteristics of C, N, and P in moss crusts and underlying soil after adding local plant biochar. It was found that biochar addition increased moss crusts and soil C, N, and P content, and changed the C: N: P stoichiometry in the moss crust-soil continuum. Additionally, there was a significant correlation between moss crust C, N, and P and soil C and N. Additionally, after adding biochar, environmental factors significantly affected the carbon, nitrogen, and phosphorus content and stoichiometric characteristics of the moss crusts. In addition, the presence of biochar strengthened the connection between moss crusts and their underlying soils, enhanced organic matter decomposition and nutrient release, and stepped up the nutrient cycling in moss crust-soil continuum. The results of this study would be helpful to understanding the nutrient dynamics of the moss crust-soil continuum and would provide a new application prospect for biochar.

## Figures and Tables

**Table 1:** The change of Stoichiometric Characteristic Values of Soil under different treatments, the data is the average value of stoichiometric characteristic values from June to October under the same treatment (mean ± SD).

Treatment	C (g kg^−1^)	N (g kg^−1^)	P (g kg^−1^)	C: N	C: P	N: P
CK	3.93 ± 0.84 d	0.5 ± 0.03 d	0.32 ± 0.022 b	7.86 ± 1.24 a	12.28 ± 1.44 d	1.56 ± 0.03 c
T1	5.69 ± 0.38 c	0.7 ± 0.02 c	0.38 ± 0.016 a	8.13 ± 0.31 a	14.97 ± 0.31 c	1.84 ± 0.04 c
T2	7.50 ± 0.52 b	0.9 ± 0.04 b	0.36 ± 0.028 ab	8.33 ± 0.21 a	20.83 ± 0.28 b	2.50 ± 0.08 b
T3	9.54 ± 0.3 a	1.2 ± 0.05 a	0.37 ± 0.022 a	7.95 ± 0.2 a	25.78 ± 0.55 a	3.24 ± 0.07 a

Notes: Different lowercase letters (a, b, c, d) indicate a significant difference (*p* < 0.05) between different biochar addition treatments.

**Table 2:** The C, N, and P characteristics of the moss crusts along biochar addition gradients, the data is the average value of stoichiometric characteristic values from June to October under the same treatment (mean ± SD).

Treatment	C (g kg^−1^)	N (g kg^−1^)	P (g kg^−1^)	C: N	C: P	N: P
CK	16.07 ± 2.20 d	0.58 ± 0.03 d	0.35 ± 0.034 d	27.71 ± 20.36 a	45.9 ± 2.94 b	1.65 ± 0.09 c
T1	38.89 ± 2.24 c	1.5 ± 0.09 c	0.53 ± 0.021 c	25.93 ± 20.57 a	73.38 ± 2.36 a	2.83 ± 0.09 b
T2	46.48 ± 2.54 b	1.8 ± 0.08 b	0.63 ± 0.041 b	25.82 ± 6.83 a	73.78 ± 2.28 a	2.86 ± 0.09 b
T3	52.79 ± 2.08 a	2.7 ± 0.09 a	0.74 ± 0.024 a	19.55 ± 3.18 b	71.34 ± 1.69 a	3.65 ± 0.04 a

Notes: Different lowercase letters (a, b, c, d) indicate a significant difference (*p* < 0.05) between different biochar addition treatments.

**Table 3:** Properties of underlying soil of moss crusts.

Treatment	Ammonium Nitrogen (mg/kg)	Nitrate Nitrogen (mg/kg)	pH	EC (ms/cm)	SMC (%)
CK	9.49 ± 1.32 c	16.41 ± 3.58 b	7.31 ± 0.12 b	0.39 ± 0.03 c	10.69 ± 1.27 c
T1	10.30 ± 1.06 bc	21.57 ± 2.91 b	7.53 ± 0.07 b	0.58 ± 0.11 b	13.06 ± 0.96 ab
T2	13.05 ± 1.18 b	23.92 ± 4.34 ab	7.80 ± 0.09 a	0.97 ± 0.09 a	12.78 ± 0.60 ab
T3	18.84 ± 2.73 a	30.06 ± 5.69 a	7.92 ± 0.1 a	1.02 ± 0.08 a	15.37 ± 2.03 a

Notes: Different lowercase letters (a, b, c) indicate a significant difference (*p* < 0.05) between different biochar addition treatments.

**Table 4:** Importance ordering and significance test of soil variables (Moss crusts).

Soil Factors	Order of Importance	F	P
Nitrate Nitrogen	1	31.83	0.001 ***
N: P	2	22.247	0.001 ***
C: P	3	19.265	0.001 ***
EC	4	9.6353	0.001 ***
pH	5	6.8974	0.009 **
SMC	6	7.1193	0.016 *
N	7	4.5511	0.03 *
P	8	2.6575	0.09
Ammonium Nitrogen	9	2.4603	0.122
C: N	10	1.7793	0.186
C	11	0.6689	0.445

Notes: The significance test of soil environmental factors * *p* < 0.05, ** *p* < 0.01, and *** *p* < 0.001.

## Data Availability

The data presented in this study are available on request from the corresponding author.
